# Spontaneous Regression of a Large Gastric Adenoma Following Gynecologic Surgery

**DOI:** 10.7759/cureus.71020

**Published:** 2024-10-07

**Authors:** Stamatina Vogli, Panagiotis Markopoulos, Aikaterini Filippakou, Gabriela Stanc, Eftychia Tsironi, Emmanouil Telakis

**Affiliations:** 1 Department of Gastroenterology, Metaxa Cancer Hospital of Piraeus, Piraeus, GRC; 2 Department of Pathology, Metaxa Cancer Hospital of Piraeus, Piraeus, GRC

**Keywords:** gastric adenoma, gastric polyp, hysterectomy with salpingo-oophorectomy, serous borderline ovarian tumor, spontaneous regression

## Abstract

Gastric adenomas are defined as polypoid lesions of neoplastic epithelium in the stomach. They are rare, occurring much less frequently than fundic gland polyps and hyperplastic polyps, and are typically associated with mucosal atrophy and intestinal metaplasia. Gastric adenomas also carry a risk of malignant transformation. We report a case of a 66-year-old woman with a gastric adenoma of the corpus found during a preoperative esophagogastroduodenoscopy before abdominal surgery for an ovarian tumor. The patient demonstrated spontaneous regression of her gastric adenoma 14 months after her initial endoscopy and subsequent hysterectomy with salpingo-oophorectomy without undergoing endoscopic resection or any other intervention. To our knowledge, this is the first well-documented case of spontaneous regression of a gastric adenoma.

## Introduction

Gastric adenomas account for 6-10% of all gastric polyps [1]. They are epithelial lesions, usually solitary, and their size ranges from a few millimeters to centimeters [2]. Endoscopically, most of them are sessile rather than pedunculated [2] and histologically, they demonstrate dysplastic changes classified as low- or high-grade dysplasia [3]. Atrophic gastritis and intestinal metaplasia are associated with the development of gastric adenomas, but a direct association with *Helicobacter pylori* infection is lacking [1,2].

Borderline ovarian tumors (BOT) are epithelial lesions with characteristics of both cystadenomas and ovarian carcinomas, representing 10-20% of all epithelial ovarian tumors [4]. Serous and mucinous BOT are the most common histologic subtypes. The presence of extra-ovarian implants represents a rare feature that worsens their generally favorable prognosis [5].

We report a unique case of spontaneous regression of a gastric adenoma following the resection of a serous BOT. To the best of our knowledge, no other case of regression of a gastric adenoma has been reported in the English literature.

## Case presentation

A 66-year-old Caucasian woman was referred by her gynecologic oncologist for a preoperative esophagogastroduodenoscopy (EGD) and colonoscopy a few days before undergoing laparotomy for suspected ovarian cancer. The endoscopies were requested in order to exclude a primary gastrointestinal tumor that might have metastasized to the ovaries (Krukenberg’s tumor) and to identify any possible colonic infiltration by the ovarian tumor. She had not undergone any previous endoscopies.

The patient had been experiencing lower abdominal discomfort for the previous two months and a physical examination revealed a palpable pelvic mass. She did not have any significant past medical history or major comorbidities. A computed tomography (CT) scan of the abdomen and pelvis followed by a magnetic resonance imaging (MRI) scan of the pelvis revealed a complex left adnexal cystic mass with a maximum diameter of 11.4 cm. Her laboratory investigations showed elevated levels of cancer antigen 125 (Ca125) (359.20 U/mL), and human epididymis protein 4 (HE-4) (170.40 pmol/L), while cancer antigen 19-9 (Ca19-9) (5.80 U/mL) and carcinoembryonic antigen (CEA) (3.25 ng/mL) were within normal limits. The rest of her laboratory findings were unremarkable. She was seen by a gynecologic oncologist and a laparotomy was scheduled.

**Figure 1:**
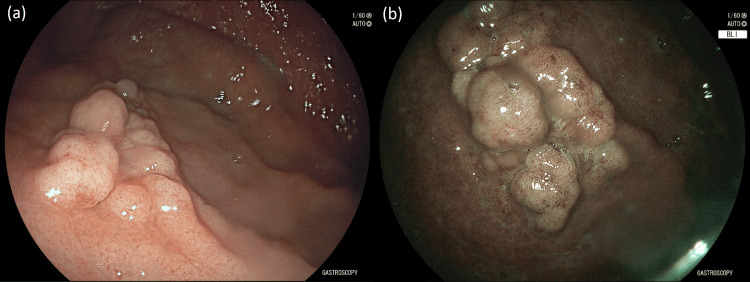
A large gastric adenoma in the proximal stomach as seen with (a) high-definition white-light endoscopy and (b) virtual chromoendoscopy using blue light imaging.

**Figure 2:**
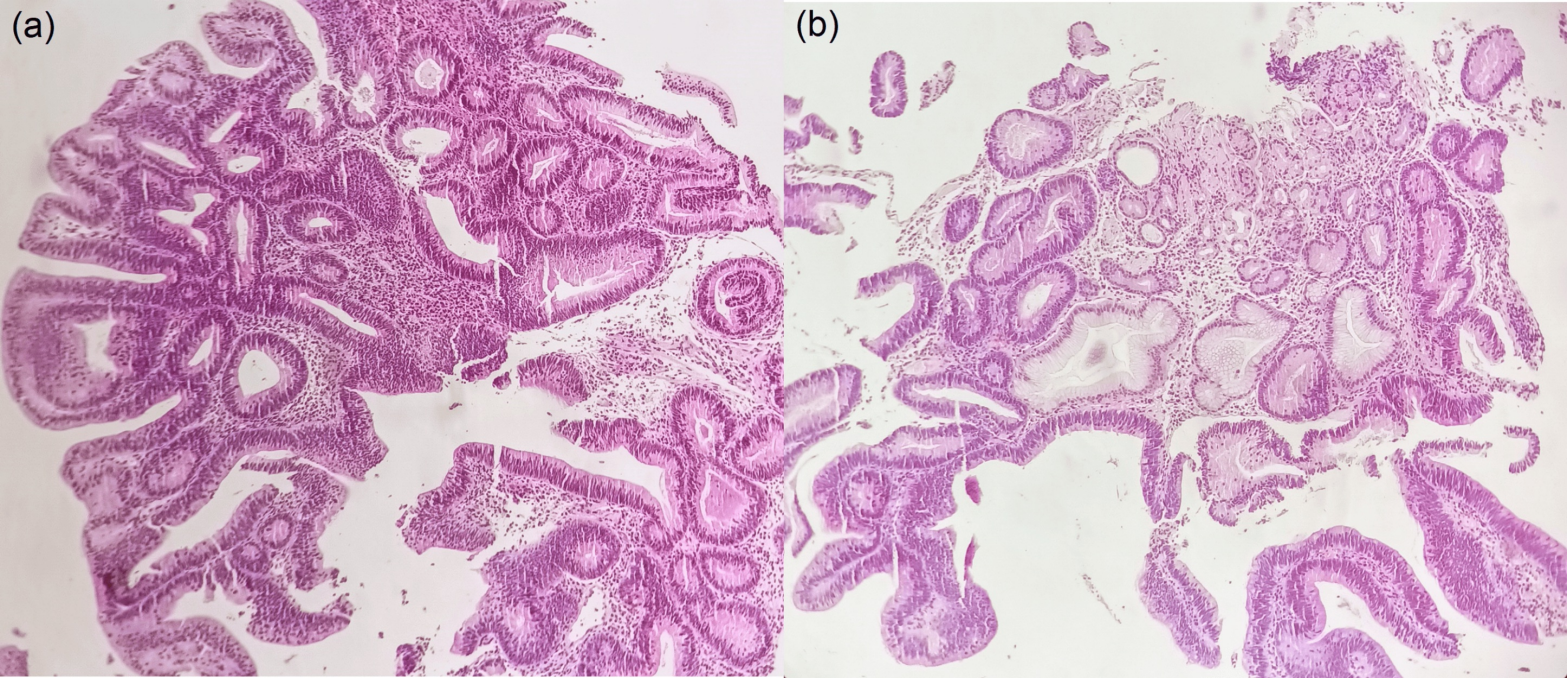
Polypoid dysplastic columnar epithelium with hyperchromatic elongated nuclei in keeping with gastric adenoma, intestinal type, with low-grade dysplasia (H&E, X100).

Her colonoscopy revealed a 20 mm pedunculated polyp in the sigmoid colon which was hot-snared. During EGD, a large polypoid lesion at the greater curvature at the junction of the body and fundus of the stomach was noted. This gastric polyp measured approximately 15x30 mm in size and was classified as a 0-Is lesion according to the Paris classification (Figure 1) [6]. Careful inspection of the polyp with high-definition white-light endoscopy (HD-WLE) and virtual chromoendoscopy using blue light imaging (BLI) did not reveal any suspicious malignant transformation areas and biopsies were taken from the lesion. Histopathology demonstrated an intestinal-type gastric adenoma with low-grade dysplasia (Figure 2) and endoscopic removal with endoscopic submucosal dissection (ESD) was recommended after her gynecologic surgery. 

The patient underwent a hysterectomy and salpingo-oophorectomy with omentum removal. Histopathological examination revealed a serous BOT with a small (0.2 mm) focal microinvasion without extra-ovarian implants (International Federation of Gynecology and Obstetrics (FIGO) stage Ia) [7]. She had an uneventful recovery and a subsequent multidisciplinary team review decided that no additional treatment was necessary and she was assigned to follow-up by her oncologist.

**Figure 3:**
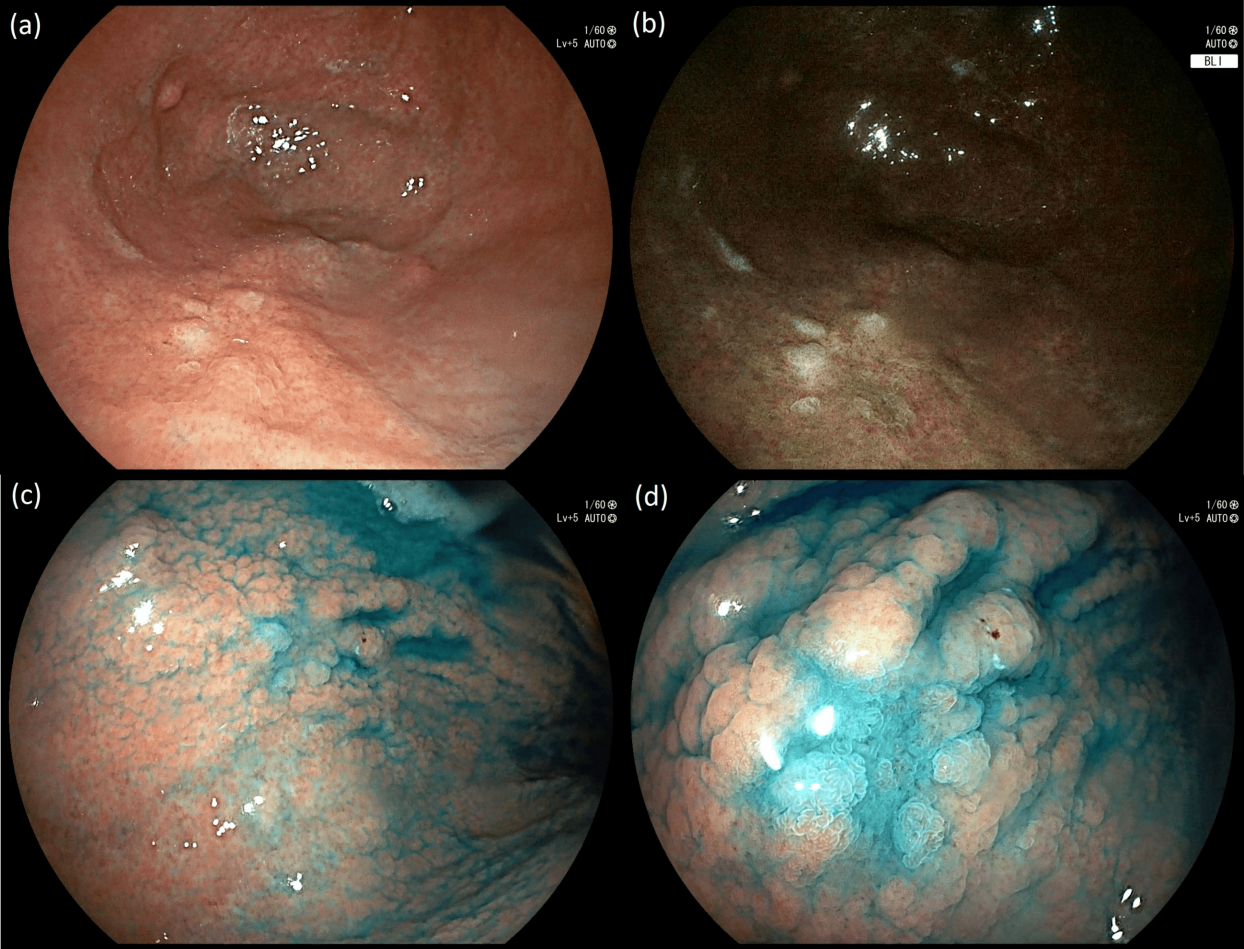
Complete regression of the gastric adenoma with a small, slightly depressed area noted at the site as seen with (a) high-definition white-light endoscopy, (b) blue light imaging, and (c, d) chromoendoscopy with methylene blue.

Unfortunately, the patient was lost to follow-up and she eventually revisited our center approximately 14 months after her initial endoscopy. Investigations did not reveal any recurrence of her ovarian cancer, and an EGD was performed to reassess the gastric lesion. Surprisingly, only a small, irregular, slightly depressed area of 15 mm in diameter was noted at the site of the gastric polyp after careful observation with HD-WLE, BLI, and chromoendoscopy with methylene blue staining (Figure 3). Biopsies from this area revealed only an edematous lamina propria with lymphocytic infiltration but no evidence of an adenoma.

**Figure 4:**
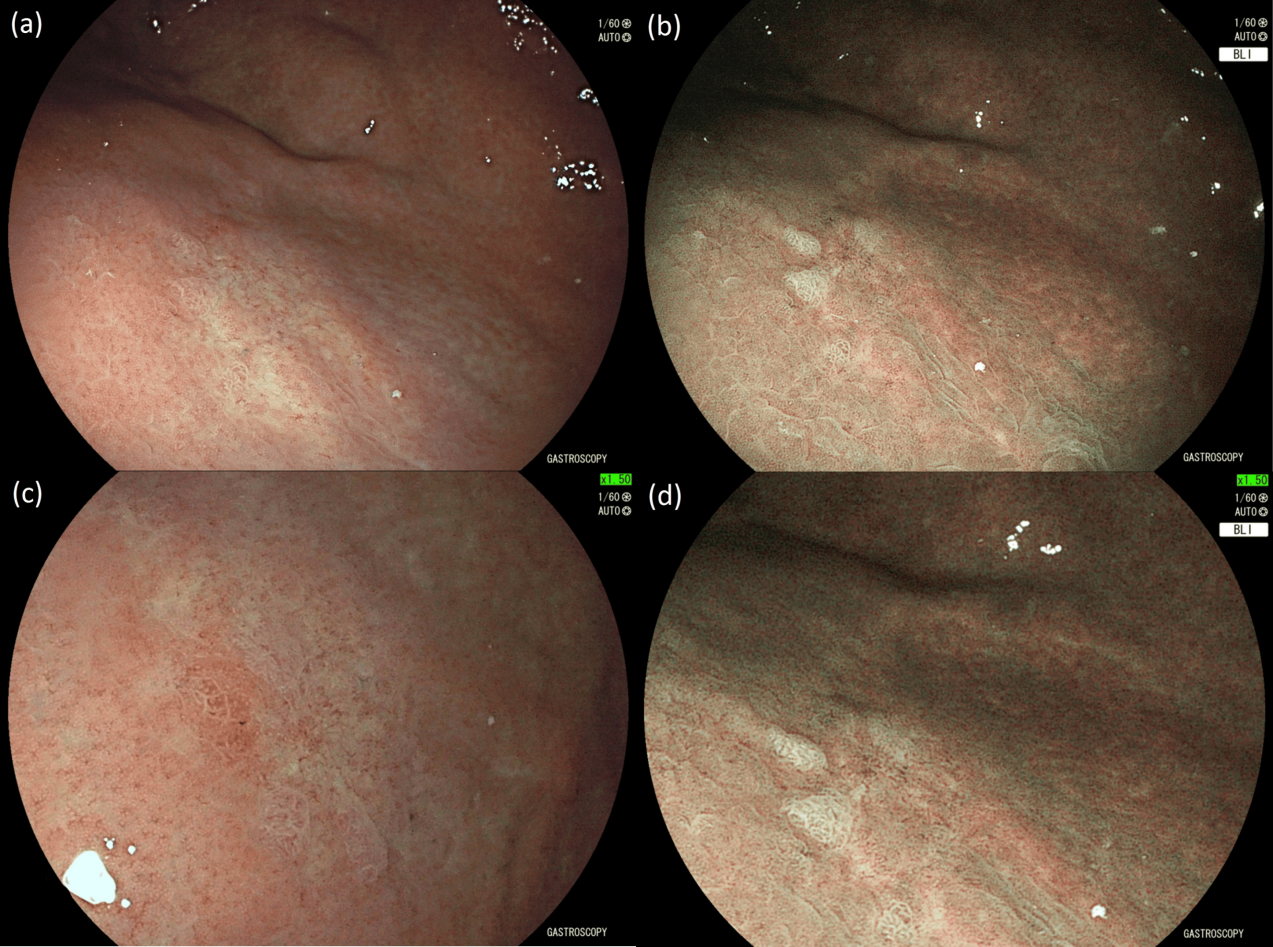
Twenty months after the index endoscopy, only a small irregular area at the site of the adenoma with irregular microvessels in the center is seen with (a) high-definition white-light endoscopy, (b) blue light imaging, (c) high-definition white-light endoscopy with electronic magnification X1.5, and (d) blue light imaging with electronic magnification X1.5.

**Figure 5:**
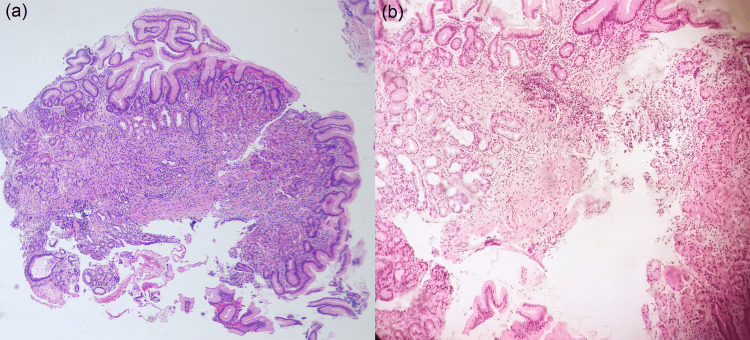
Atrophy of gastric mucosa with focal pseudopyloric metaplasia and a mixed chronic inflammatory infiltrate; (a) H&E X40, (b) H&E X100.

Due to these unexpected findings, another EGD was performed six months later which again did not reveal any evidence of the gastric polyp. The same small irregular area was noted at the site with irregular microvascular patterns seen in the center using HD-WLE and BLI with electronic magnification (Figure 4). Biopsies showed atrophy of the gastric mucosa with focal pseudopyloric metaplasia and mixed chronic inflammatory infiltrate (Figure 5). Biopsies from the antrum and body were unremarkable apart from evidence of intestinal metaplasia in the antrum. Immunohistochemical staining for *H. pylori* infection was negative in all specimens. According to European guidelines for gastric epithelial dysplasia surveillance [2], the patient was scheduled to undergo an EGD with high-definition chromoendoscopy in one year. 

## Discussion

We present an extremely unusual case of spontaneous regression of a gastric adenoma following the resection of a serous BOT. Gastric adenomas with either low- or high-grade dysplasia are classified as neoplastic lesions and are considered precursors to gastric cancer [1]. Nearly half of those measuring more than 20 mm in size may contain foci of adenocarcinoma and the coexistence of cancer elsewhere in the gastric mucosa has been reported in up to 30% of such cases [2,8]. For these reasons, gastric adenomas should be endoscopically resected en bloc, with ESD being the recommended excision method for sessile lesions larger than 15 mm [1,2]. A careful evaluation of the stomach is recommended to identify synchronous neoplasia, atrophy, or intestinal metaplasia while surveillance EGD after resection should be performed at intervals indicated by the number, size, and histologic characteristics of the polyps [2].

Our literature review did not identify any other reported case of spontaneous regression of a gastric adenoma. Although the disappearance of a polypoid lesion can sometimes be explained in terms of tumor dislodgement [9], it would seem an unlikely mechanism in the present case since the polyp lacked a stalk. Gastric hyperplastic polyps, which are benign gastric lesions strongly associated with *H. pylori* infection, frequently regress following *H. pylori *eradication [10].

Few reports of spontaneous regression of other benign gastrointestinal tumors have also been published [9] but never regarding a gastric adenoma. Spontaneous regression of cancer, defined by Stewart [11] as the partial or complete disappearance of a malignant tumor in the absence of treatment, has been reported with the first case described over 70 years ago; however, it is an exceedingly rare phenomenon. According to the review by Minacappelli et al., 289 cases of primary gastrointestinal carcinomas were reported to exhibit spontaneous regression during the previous century with eight cases among them being gastric cancers [9].

The specific mechanisms underlying these phenomena have not been elucidated yet and most of the proposed explanations are derived from observations of small series and cases of spontaneous cancer regression as data for benign epithelial tumors are scarce. According to the review by Radha et al., immunological responses of the host along with ischemic models account for the majority of the possible explanations of spontaneous gastrointestinal neoplasia regression [12]. Immune cell infiltration of the tumor and the release of interleukins (IL), specifically IL-2, IL-6, and IL-8, tumor necrosis factors, and interferons have proven their immunomodulatory effects in a number of studies [12,13]. Hypoperfusion and ischemia, either systemic or local, could also play a role considering the increased blood supply needs of a neoplastic tumor [14,15]. Hormonal factors, epigenetic changes, and alterations in circulating cytokines, growth factors, and tumor suppressors are among the proposed mechanisms, as well [12].

Ovarian tumors, both malignant and benign, are linked with paraneoplastic syndromes which are induced by substances secreted by the tumor cells or by an immune response of the host and can affect most of the human body systems [16]. Characteristically, a case of Zollinger-Ellison syndrome induced by a BOT has been described in the literature [17]. The tumor microenvironment, composed of the tumor cells, their associated cells, and a variety of secreted molecules, is thought to communicate with distal tissues through circulating substances and extracellular vesicles in both endocrine and paracrine fashions [18]. Growth factors, hormones, and cytokines can be released into the circulation forming a molecular cross-talk that is poorly understood so far. Data suggest that benign ovarian tumors secret growth factors such as epidermal growth factor (EGF), transforming growth factor (TGF), and vascular endothelial growth factor (VEGF) [19] whose receptors are often overexpressed in solid neoplastic tumors. The expression of VEGF and IL-6 has been found up-regulated in gastric adenomas [20]. A better understanding of the above-mentioned molecular signaling pathways may offer insight into the largely unknown pathophysiologic mechanisms of spontaneous tumor regression. In our case, the surgical removal of the ovarian tumor may have played a role in the regression of the gastric adenoma by downregulation of these circulating factors and substances.

## Conclusions

To our knowledge, this is the first well-documented case of spontaneous regression of a gastric adenoma reported in the English literature. Cases of spontaneous regression of other benign and malignant gastric tumors have been described but the exact pathophysiologic mechanisms behind this phenomenon remain unclear so far. In our case, there is a plausible connection between the surgical removal of the BOT and the regression of the adenoma, suggesting that the elimination of circulating substances produced by the tumor and its microenvironment may have played a role. Further research is necessary to investigate the potential mechanisms involved in the spontaneous regression of gastric tumors.
